# Sodium Hypochlorite/Amino Acid Gel in the Non-Surgical Treatment of Periodontitis—Clinical and Molecular Results of Randomized Clinical Trial

**DOI:** 10.3390/jfb16120470

**Published:** 2025-12-18

**Authors:** Ewa Dolińska, Katarzyna Golińska, Violetta Dymicka-Piekarska, Robert Milewski, Magdalena Sulewska, Małgorzata Pietruska

**Affiliations:** 1 Department of Periodontal and Oral Mucosa Diseases, Medical University of Bialystok, ul. Waszyngtona 15, 15-269 Bialystok, Poland; magdalena.sulewska@umb.edu.pl (M.S.);; 2 Private Practice, ul. Rybacka 24A/1, 12-200 Pisz, Poland; k.guminska@wp.pl; 3 Department of Clinical Laboratory Diagnostics, Medical University of Bialystok, ul. Waszyngtona 15, 15-269 Bialystok, Poland; violetta.dymicka-piekarska@umb.edu.pl; 4 Department of Biostatistics and Medical Informatics, Medical University of Bialystok, ul. Szpitalna 37, 15-295 Bialystok, Poland; robert.milewski@umb.edu.pl

**Keywords:** scaling and root planning, antiseptic, low concentration hypochlorite gel, nonsurgical periodontal therapy, periodontitis, periodontal therapy

## Abstract

Due to the limitations of SRP, new methods are being sought to support non-surgical periodontal therapy. One of them is the use of antiseptics such as low-concentration sodium hypochlorite gel buffered with amino acids (NaOCl/AA). The aim of the study was to evaluate periodontal parameters and the concentration of metalloproteinase 8 (MMP-8) and interleukin 8 (IL-8) in the gingival crevicular fluid (GCF) after SRP with or without NaOCL/AA gel. The study included 40 periodontal patients randomized to study and control groups. Before SRP, the study group had a gel introduced into pockets with PD ≥ 5 mm. After treatment in both groups, the pocket depth (PD) decreased, there was a CAL gain, and unnoticeable changes in the gingival recession (GR). In the study group, deep pockets accounted for 25% of the sites examined prior to therapy, whereas after therapy, they decreased to 12%. In the control group, the proportion of deep periodontal pockets (PD ≥ 5 mm) fell from 17.46% to 9.05%. No differences were noted between groups. In the study group, there was a significant reduction in the amount of MMP-8 in GCF from 8.32 ng/mL to 5.14 ng/mL after 3 months. No statistically significant difference was observed in the control group. The concentration of IL-8 decreased significantly over time in both groups without differences between them. A single application of the NaOCl/AA gel in deep periodontal pockets does not affect clinical results and IL-8 levels. However, it had a significant effect on the amount of MMP-8.

## 1. Introduction

Periodontitis is a chronic inflammatory disease leading to bone and soft tissue loss and, consequently, tooth loss [1]. The incidence of periodontal disease increases with age and rises sharply in people aged 50–60 years. The proportion of people with periodontitis is expected to continue to increase due to the ageing population [2].

The primary treatment modality of periodontitis is non-surgical periodontal therapy—the SRP (scaling and root planing). SRP is a causal therapy that reduces inflammation, decreases probing depth, and allows a gain in the clinical attachment. The effectiveness of the SRP depends on many factors, including the initial pocket depth, the type and shape of the tooth, the furcation involvement, and the experience of the operator. Due to the limitations of SRP and the multifactorial etiology of periodontitis, methods are being sought to support mechanotherapy. One is the use of antiseptics, especially iodopovidone, diluted sodium hypochlorite, or chlorhexidine [3]. The current guidelines of the European Federation of Periodontology also allow for the additional use of antiseptics as a supplement to mechanical debridement during step 2 and supportive periodontal therapy in the treatment of stage I-III periodontitis [4]. Sodium hypochlorite has many characteristics that make it useful in periodontics. These include strong bactericidal activity, relatively low toxicity at the recommended concentrations, lack of colour, not causing tooth discolouration, easy availability, and low cost. At low concentrations, sodium hypochlorite is used as a topical antimicrobial agent to clean wounds and skin ulcers without inhibiting fibroblast function [3,5]. A new two-component preparation containing 0.95% NaOCl and amino acids (glutamic acid, leucine, and lysine) is used in periodontics. When the two components of the preparation are mixed, short-acting chloramines are obtained. Chloramines have bactericidal, virucidal, fungicidal, and protozoal effects [6]. Ready-to-use gel contains 0.5% sodium hypochlorite.

Extracellular matrix metalloproteinases (MMPs) are a family of enzymes that degrade extracellular matrix and basal lamina components. Their expression in mature tissues is relatively low, but increases during inflammatory diseases, tumour growth, or metastasis. This can lead to unwanted tissue destruction, as occurs during periodontal disease [7]. MMP-8 (collagenase-2) is the main collagenolytic enzyme in gingival pocket fluid (GCF) and saliva. MMP-8 breaks down gingival connective tissue and is responsible for 90–95% of collagenolytic activity in GCF. Collagenase activity in GCF and MMP-8 concentrations were found to correlate with the amount of type I collagen breakdown products in sites with active periodontitis compared with inactive sites [8]. Conventional, causal periodontal treatment (SRP) has been shown to reduce the amount of MMPs in saliva [9] and GCF [10,11]. However, in patients with periodontitis, even after successful therapy, the amount of MMP-8 in the pocket remains higher compared to healthy subjects [12]. This indicates an increased local host response to inflammation. Therefore, MMP-8 levels may be a marker of health, disease, or the effectiveness of the periodontal therapy used.

IL-8 is a potent chemoattractant for neutrophils and a proinflammatory cytokine in periodontal disease. Macrophages, fibroblasts, and epithelial cells produce IL-8 in response to microbial invasion. It increases neutrophil recruitment from the highly vascular gingival tissue to the gingival crevicular fluid [13,14].

Still, not much is known about clinical and molecular periodontal disease indicators before and after SRP with adjunctive use of chloramine-based antiseptics.

In view of the above, the present study aimed to assess clinical periodontal parameters and metalloproteinase 8 (MMP-8) and interleukin 8 (IL-8) concentrations in GCF after scaling and root planning with and without the single subgingival application of a low-concentration sodium hypochlorite buffered with amino acids gel (NaOCl/AA).

## 2. Materials and Methods

### 2.1. Study Design and Study Group

The study was designed as a randomized, controlled clinical trial. Forty adult patients with periodontitis aged between 29 and 67 years (32 females and 8 males) were enrolled to the study. Eligibility criteria for participation were diagnosed periodontitis stage II or III, grade B or C [4]; presence of at least 16 teeth (at least 4 in each quadrant); presence of at least 4 deep periodontal pockets (PD ≥ 5 mm); no professional hygiene procedures in the last 6 months; no systemic antibiotic therapy in the last 3 months; age over 18 years; being non-smoker. Exclusion criteria for participation in the programme were: general contraindications to any periodontal therapy; immunosuppression; immunological incompetence; uncontrolled diabetes; pregnancy and breastfeeding; alcohol and/or drug dependence; patient requiring antibiotic cover prior to periodontal therapy; patient with no opportunity to participate in the programme for 6 months.

Patients were randomly allocated to the study or control group (20 patients each). Randomization was based on a computer-generated list. Sample size calculation was performed a priori, assuming a standard deviation of CAL change of 1 mm and to detect a mean difference of 1 mm with a test power of 80% on 32 subjects. However, we considered possible drop-outs, so we decided to recruit and randomize 40 patients. The study was conducted at the Department of Periodontal and Oral Mucosal Diseases, Medical University of Bialystok, from January 2021 to March 2022. The study was in accordance with the Helsinki Declaration of 1975, updated in 2000, and approved by the Bioethics Committee of the Medical University of Bialystok (APK.002.269.2020). Each subject was informed about the study and signed a written consent. The study was registered under ISRCTN number 11287170.

### 2.2. Analysis of Clinical Parameters

A detailed clinical periodontal examination and the collection of gingival crevicular fluid (GCF) from a selected pocket (PD ≥ 5 mm) were performed before treatment and 3 and 6 months after. Additionally, 1 week and 2 weeks after treatment, GCF was collected from the pocket selected at the first visit, and patients were re-motivated to maintain optimal oral hygiene. The clinical examination consisted of an assessment of probing depth (PD), gingival recession (GR), clinical attachment level (CAL), number of deep periodontal pockets, i.e., pockets with PD ≥ 5 mm, full-mouth plaque score (FMPS), and full-mouth bleeding on probing (FMBOP). A manual PCP UNC 15 periodontal probe (Hu-Friedy, Chicago, IL, USA), calibrated in 1 mm increments, was used for clinical examination.

### 2.3. Clinical Procedure

Patients randomized to the study group were treated non-surgically (SRP, scaling and root planing) according to the following procedure: after a periodontal examination, a preparation with NaOCl/AA (Perisolv^®^, Regedent AG, Zurich, Switzerland) was applied into the pockets with PD ≥ 5 mm. The formulation was prepared immediately before application by mixing the contents of two syringes. A gel was introduced into the pocket with PD ≥ 5 mm with a sterile applicator. The gel was left in place for 30 s. This was followed by a total subgingival scaling and root planing using an EMS Piezon ultrasonic scaler (EMS, Electro Medical Systems, Nyon, Switzerland) with a PS tip (Perio Slim). In the control group, the SRP procedure was performed with the same instruments without Perisolv^®^ application. The clinical examination was conducted by one blinded and calibrated examiner (K.G.), and periodontal treatment by one experienced periodontist (E.D.).

### 2.4. Collection of Material for Laboratory Analysis

Before therapy, 1 week, 2 weeks, 3, and 6 months after treatment, GCF was collected from a pocket (PD ≥ 5 mm) selected at the first visit (the only criterion for selecting pockets for GCF collection was their baseline depth). The fluid was collected using sterile PerioPaper Strips (Interstate Drug Exchange, Amityville, NY, USA). Prior to the procedure, the tooth was isolated from saliva and dried. A PerioPaper Strip was gently placed 1–2 mm deep in the pocket and left for 30 s to absorb the fluid. Strips contaminated with blood were discarded. The amount of gingival fluid from the pocket- SFFR (sulcus fluid flow rate) in relative Periotron units (PU- Periotron units) was then measured using a Periotron 8010 device (Oraflow Inc., Plainview, NY, USA). The strip was placed in Eppendorf Safe-Lock Tubes 2.0 mL (Eppendorf, Hamburg, Germany) with 200 μL phosphate-buffered saline (PBS, phosphate-buffered saline) for 15 min. After 15 min, the tubes were placed in a vortex MX-S shaker (DLab Scientific Inc., Rowland, CA, USA) for 15 s. The paper strip was removed from the tube to avoid contamination. Immediately after collection, samples were frozen until laboratory analysis.

### 2.5. Laboratory Procedure

GCF collected from a selected pocket with PD ≥ 5 mm was analyzed in the laboratory. The metalloproteinase 8 (MMP-8) content was assayed using ready-to-use ELISA kits (Human MMP-8 Elisa kit, R&D Systems, Minneapolis, MN, USA) and IL-8 (Human IL-8/CXCL8, 8 Elisa kit, R&D Systems, Minneapolis, USA) according to the manufacturer’s instructions. Results were presented as the amount of fluid collected per 30 s per measuring point and expressed in ng/mL (MMP-8) and pg/mL (IL-8). Lamster et al. stated that presenting data as concentration may not be appropriate for GCF because this leads to the assumption that each sample contains the same amount of fluid, which is representative of the total volume of fluid [15]. Representing the results as the total amount of enzyme per 30 s sample eliminates errors from the analysis [16].

### 2.6. Statistical Analysis

Due to the inability to verify the normality of the distribution and the relatively small size of the groups, non-parametric tests were used in the statistical analysis. Comparing quantitative variables, the non-parametric Mann–Whitney U test was used for two groups. Comparing dependent variables, the Friedman ANOVA test was used for multiple variables. Spearman’s rank correlation coefficient was also determined. Statistically significant results were considered at *p* < 0.05. Statistica 13.3 (Tibco Software Inc., Palo Alto, CA, USA) was used in the calculations.

## 3. Results

### 3.1. Study Participants

**Figure 1:**
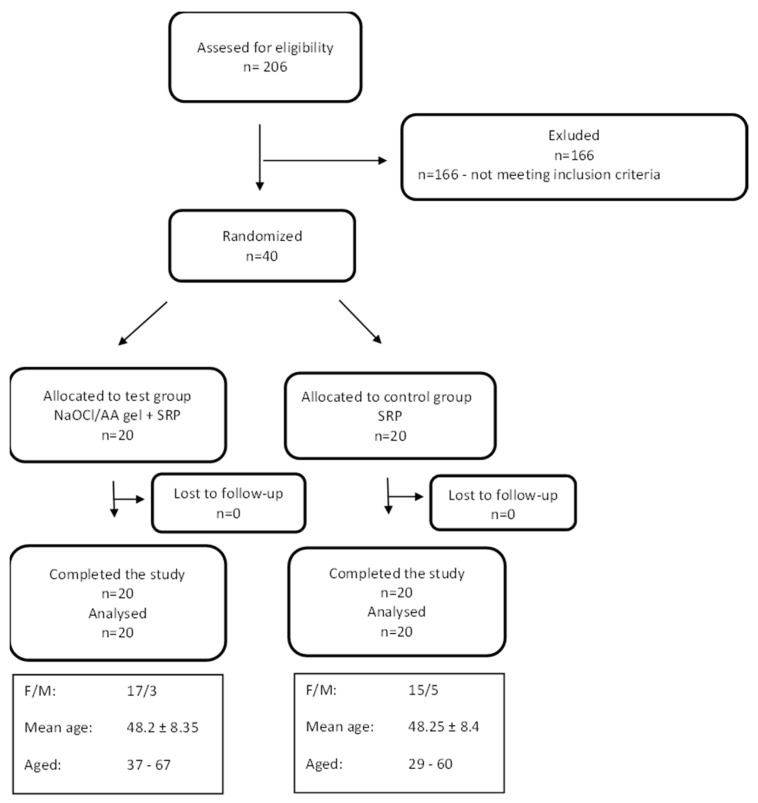
CONSORT flow-chart of the study and study patient characteristics.

Forty patients meeting the inclusion criteria were recruited for the study. No adverse events were reported during the 6-month follow-up period. All patients attended follow-up visits. Patients were allocated to the study and control groups based on a computer-generated randomisation list. The process of recruitment, follow-up, and analysis of patient data is shown in the Consort diagram (Figure 1).

### 3.2. Changes to FMPS and FMBOP

Prior to therapy, patients had moderate oral hygiene expressed as FMPS values above 20% and an inflammation index (FMBOP) close to 29% in the treatment group and 19% in the control group. Table 1 compares the mean values of FMPS and FMBOP in the observation period. In both groups, FMPS decreased (without significance), as well as the number of bleeding sites (with significance).

### 3.3. Changes in Mean PD, GR, CAL for the Entire Dentition

**Figure 2:**
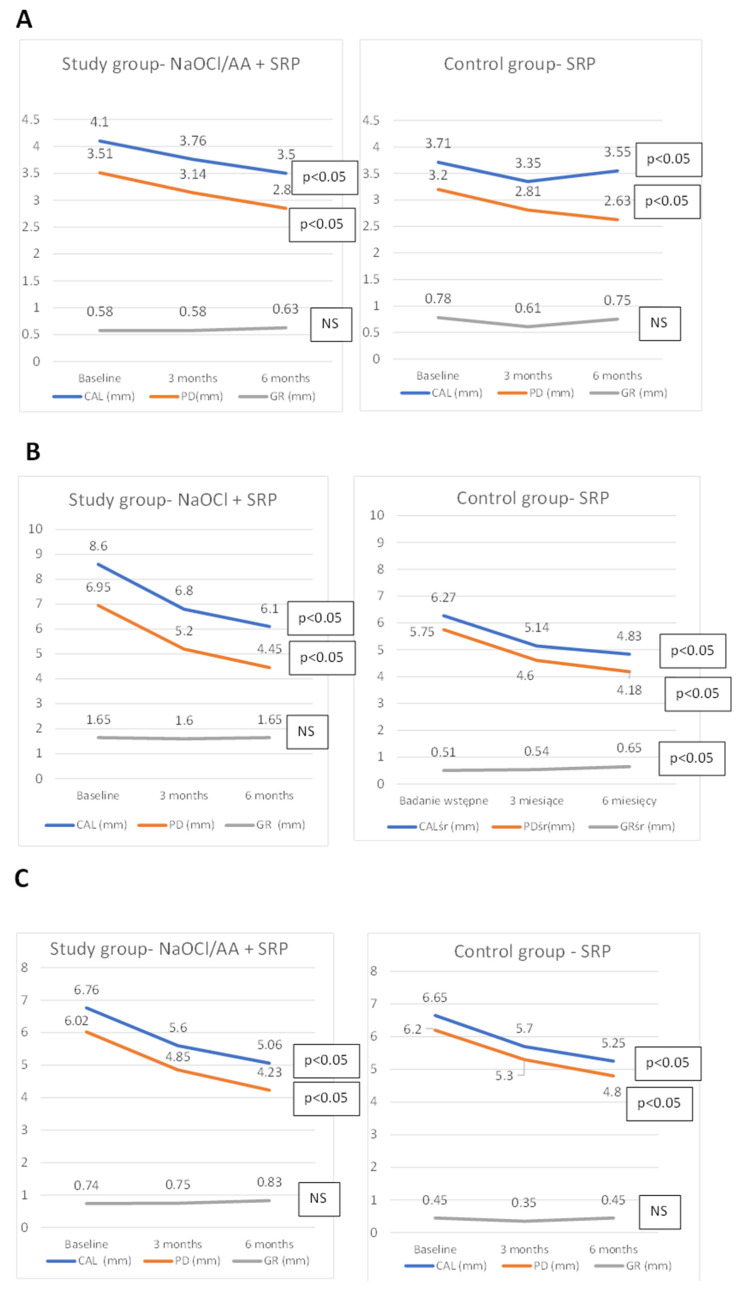
Changes in mean periodontal parameters (PD, GR, CAL) for the entire dentition (**A**), pockets PD ≥ 5 mm (**B**), pocket chosen for immunological analysis (**C**).

In both the control and the study group a reduction in the probing depth was achieved. There was also a significant difference in the clinical attachment level. CAL gain was found in both groups. The CAL gain in the study group averaged 0.6 mm and in the control group 0.15 mm. Changes in the height of gingival recessions in both groups were very small. The exact data are included in Figure 2 and Table A1.

### 3.4. Changes in Mean PD, GR, CAL of Deep Pockets

Comparing the means of the periodontal parameters studied (PD, GR, CAL) only from pockets with an initial PD ≥ 5 mm, there was a PD reduction and a CAL gain over six months in both groups. The GR increased in both groups. However, only significant for the control group. Reduction in the pockets depth in the study group was 1.8 mm, and CAL gain was 1.7 mm; in the control group, it was 1.57 mm and 1.43 mm, respectively. No differences were noted between the groups (Figure 2, Table A2).

### 3.5. Analysis of the Number of Pockets with PD ≥ 5 mm

**Figure 3:**
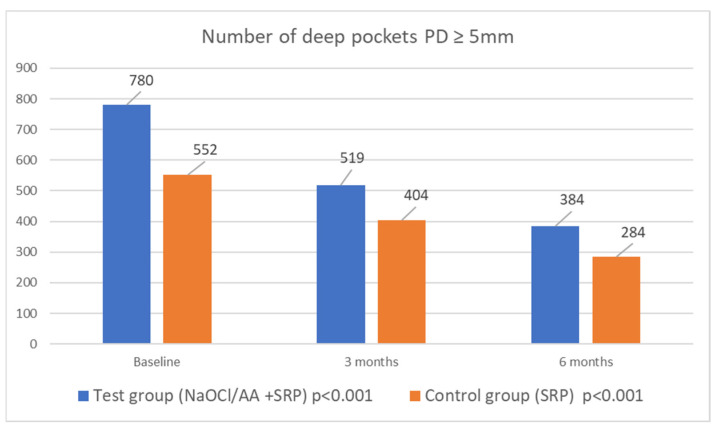
Number of deep periodontal pockets (PD ≥ 5 mm) in both groups in 6 months observation time.

In patients of the study and control groups, the mean number of deep pockets decreased significantly over time. In the study group before therapy, pockets with PD ≥ 5 mm accounted for almost 25% of the sites probed; after therapy with NaOCl/AA gel, this was only 12%. In the control group, the percentage of deep periodontal pockets decreased from 17.46% to 9.05%. No differences were noted between the groups. In the study group, 396, and in the control group, 268, pockets were ‘closed’. Data relating to the number of pockets with PD ≥ 5 mm are presented in Figure 3.

### 3.6. Analysis of Clinical Parameters (PD, GR, CAL), Pocket Fluid Volume (SFFR), and MMP-8 and IL-8 Concentrations for the Selected Periodontal Pocket

In addition, one pocket was selected in each patient to collect fluid from the pocket (GCF) and determine changes in the amount of MMP-8 and IL-8. The changes in clinical parameters of the selected pocket are shown in Figure 2 and Table A3. In the study group, there was a significant reduction in the amount of MMP-8 in the selected pocket from 8.32 ng/mL to 5.14 ng/mL after 3 months. No significant difference was noted in the control group. The concentration of MMP-8 differed between the groups before the study. The concentration of IL-8 decreased significantly over time in both groups, with no difference observed between them. Changes in the amount of SFFR (sulcus fluid flow rate) were unremarkable in both groups. Changes in the SFFR, MMP-8, and IL-8 are shown in Table 2.

### 3.7. Spearman’s Rank Order Correlations

The correlations between the volume of fluid collected from the selected pocket and the concentrations of MMP-8 and IL-8 were also examined. Additionally, on the baseline and after three months, the correlation between the number of deep periodontal pockets and FMPS and FMBOP was checked. In the study group, strong correlations were observed between MMP-8 and IL-8 concentrations before therapy, one week, and three months afterwards. In the control group, MMP-8 and IL-8 correlated with each other in all study points except the one after a week. No relationship was found between the number of persistent pockets and FMPS or FMBOP in the study group. However, the control group showed a relationship between FMBOP and the number of pockets with PD ≥ 5 mm before therapy and three months after therapy. The correlation data are presented in Table 3.

## 4. Discussion

The aim of the study was to compare the effectiveness of basic nonsurgical therapy (SRP) with SRP enriched with additional application of a low-concentration sodium hypochlorite/amino acids gel into deep periodontal pockets. The reduction in probing depth (PD) and the number of ‘closed’ pockets were considered the primary parameters in the analysis. Clinical attachment level gain (CAL), gingival recession (GR), full mouth plaque score (FMPS), and full-mouth bleeding on probing (FMBOP) were chosen as secondary study determinants. Changes in GCF concentration of metalloproteinase 8 (MMP-8) and interleukin 8 (IL-8) were analyzed as additional laboratory indicators.

Patients enrolled in the study had moderately good oral hygiene. In both groups, the pre-treatment FMPS rate was over 25% and had decreased, without differences between groups. The FMBOP objectively reflects periodontal inflammation and is routinely used in periodontal practices to assess the course of inflammation and monitor ongoing therapy. The absence of bleeding on probing is a reliable indicator of the stability of the periodontal condition [17]. Achieving FMBOP < 15% is one of the main objectives of periodontal therapy. An FMBOP value < 15% allows the qualification of the patient for periodontal surgery or transition from the active phase of treatment to the maintenance phase, when a goal of PD < 6 mm is concomitantly achieved [17,18,19]. According to present guidelines, a stable periodontitis patient is defined as someone who has completed periodontal therapy and has gingival health on a reduced periodontium (bleeding on probing in less than 10% of sites, with shallow probing depths of 4 mm or less, and no sites with bleeding on probing at 4 mm). However, if these criteria are met but bleeding on probing is present at >10% of sites after the completion of periodontal treatment, the patient is diagnosed as having stable periodontitis with gingival inflammation [4]. Patients in both the study and control groups had a BOP of over 15% prior to the commencement of therapy. After six months, the FMBOP had decreased significantly in both groups, to 14.32% in the study group and 14.35% in the control group. The goal of reducing inflammation was achieved in both groups, with no difference between them.

After 6 months, a statistically significant shallowing of periodontal pockets in both groups was observed. In the study group, the average PD was reduced from 3.51 mm to 2.85 mm (0.67 mm reduction), and in the control group from 3.2 mm to 2.63 mm (0.57 mm reduction). The analysis carried out for initially deep pockets (with PD ≥ 5 mm) also showed a significant reduction in PD. These pockets were treated with a NaOCl/AA gel in the study group. Thus, in the study group, the average PD of deep pockets was reduced from 6.02 mm to 4.23 mm (a reduction of 1.8 mm), and in the control group from 5.75 mm to 4.18 mm (a reduction of 1.57 mm). These reductions were significant over time for each group, but there were no differences between groups in relation to PD. Clinical attachment level (CAL) gain was observed in both groups and was significant over time when considering all measurement points, as well as those associated with deep pockets. In both cases, there were no differences between the groups in relation to CAL.

In the course of periodontal treatment, it is also important to eliminate pockets with a PD ≥ 5 mm. Deep pockets provide a favourable environment for the growth of anaerobic bacteria, including periopathogenic Gram-negative bacteria [20]. Probing depth reduction and a decrease in the number of pockets with a PD ≥ 5 mm create the right conditions for the development of symbiotic flora. In the study group, 396, and in the control group, 268 pockets „were closed”. At baseline, 24.65% of the pockets in the study group were deep; after treatment, this indicator fell to 12.05%. In the SRP group, the proportion of deep pockets decreased from 17.46% to 9.05%. This demonstrates the very good results of the treatment and is consistent with the results of a systematic review, which looked at pocket closure and residual pockets after non-surgical therapy [21]. In our study, 12.05% of probing sites in study group were residual pockets at six months post-treatment, compared to 9.05% in the control group. However, the results of the cited systematic review show that after non-surgical treatment, up to 14.13% of pockets with PD ≥ 5 mm remain [21].

The efficacy of non-surgical periodontal therapy in patients with periodontitis is well documented in the literature [22,23,24], and SRP itself continues to be the gold standard in the treatment of patients with periodontal disease. However, there is a need to develop methods that support SRP without causing side effects. The low-concentration sodium hypochlorite buffered with amino acids may have such properties, which is why it has been extensively studied. The study that is most similar to ours was conducted by Iorio-Siciliano et al. [25]. In this study, patients with untreated periodontitis at moderate risk of progression (grade A/B) were excluded. For this reason, the average pocket depths were much higher than in our study, as our eligible patients had intermediate periodontitis (stage II/III, grade B/C). In the study group led by Iorio-Siciliano, the pockets shallowed by 2.49 mm, whereas in the control group, the reduction in PD was 1.98 mm. In our study, PD decreased by 0.67 mm in the test group and by 0.57 mm in the control group. The patients in our study had less advanced disease, which is why the reductions were smaller. The results, in the cited study, were in favour of the study group according to CAL. In our case, the differences between the groups did not reach statistical significance. The Italian researchers achieved a significant quantitative reduction in the number of pockets with PD ≥ 5 mm. The number of such pockets decreased from 763 to 20 in the amino acid-buffered sodium hypochlorite gel group, and from 594 to 53 in the control group. In our study, these reductions were 780 to 384 and 552 to 284, respectively, in the study and control groups. These promising results allowed the Italian researchers to conclude that using NaOCl/AA gel together with the SRP procedure shows significantly greater clinical improvement than using SRP alone [25]. Despite a satisfactory improvement in clinical parameters, our study did not demonstrate significant differences between the groups. Similar conclusions to ours were drawn by Megally et al. [26]. The cited researchers studied the efficacy of SRP treatment with NaOCl/AA gel and SRP treatment alone at one-year follow-up in patients undergoing maintenance periodontal treatment. The study group experienced a reduction in deep periodontal pockets of 0.97 mm, while the control group experienced a reduction of 0.85 mm. CAL gain was 1.02 mm and 0.82 mm, respectively. After twelve months, 47% of pockets with PD ≥ 5 mm remained in the study group, compared to 49% in the control group. There were no significant differences between the two groups, suggesting that there was no major benefit from the topical application of NaOCl/AA gel [26]. The results of the cited papers are not consistent. The discrepancy in results may be explained by the fact that pockets previously untreated respond more favourably to SRP than persistent pockets present in the maintenance phase. Patients with untreated periodontitis have deeper pockets and more pockets with subgingival deposits and bacterial biofilm than patients on maintenance therapy. Therefore, sodium hypochlorite gel achieves better clinical results in untreated patients. Radulescu et al. also evaluated the use of NaOCl/AA gel in maintenance therapy and compared it to 1% gel with CHX and placebo. Of the sites with NaOCl/AA gel, 77.5% of the pockets were ‘closed’. The reduction in probing depth was greater in the CHX group. However, the NaOCl/AA gel was more effective than the placebo. After six months, CAL gain was higher in the group with NaOCl/AA gel than CHX. It was concluded that NaOCl/AA gel may also benefit patients on maintenance therapy [27]. Diehl et al. carried out an analysis of a case series in which persistent periodontal pockets were cleaned with NaOCl/AA gel, followed by application of hyaluronic acid. After 6 months, the reduction in PD as well as CAL gain was 2 mm. There was also a 60% reduction in BOP and closure of 25% of deep pockets. These are promising results from the use of sodium hypochlorite and hyaluronic acid preparation together with SRP [28]. This combination is increasingly reported in the literature with promising clinical results [29,30].

A collection of GCFs allows quantitative and qualitative assessment of the sample. The volume of GCF increases with the severity of inflammation in the pocket [31], and GCF taken from a specific measuring site provides an excellent means of assessing the biological processes that occur in the periodontium. Healing after SRP treatment takes up to three months [32], with the greatest periodontal changes occurring immediately after treatment. Therefore, pocket fluid was collected at 1 and 2 weeks after SRP treatment and at 3 months.

MMP-8 was chosen for the assay because it is one of the best-described biomarkers of periodontal disease, and, since 2005, there has been a growing body of evidence supporting the diagnostic value of MMP-8 [33]. A higher amount of MMP-8 has been reported in GCF taken from patients with inflamed tissues [34,35], and the amount of active MMP-8 is significantly related to pocket depth [36]. Periodontal therapy has been described to cause a decrease in the amount of collagenase 2 in the GCF of patients with periodontal disease [37,38,39]. Thus, collagenase 2 concentration may be an additional immunological marker of the efficacy of the therapy. In our study, the concentration of MMP-8 decreased statistically in the group where NaOCl/AA gel was applied, dropping from 8.32 ng/mL to 5.14 ng/mL after three months, with the lowest value recorded one week after therapy. The results were completely different in the control group, where the SRP was followed by a slight increase in MMP-8 concentrations, which returned to a level close to the baseline after 3 months. This may indicate the efficacy of the tested preparation. However, these results are inconclusive due to statistical differences observed between the groups at baseline. Therefore, confirmation of this result requires further research.

By its very nature, IL-8 is of great interest for a better understanding of the mechanisms leading to neutrophil-mediated tissue destruction [40]. A recent study showed a significant reduction in salivary levels of pro-inflammatory cytokines such as IL-1β, IL-6, and IL-8 in patients who underwent non-surgical periodontal therapy [41]. The decrease in IL-8 levels in GCF after SRP was also confirmed [42]. We observed a significant drop in IL-8 concentration in both groups after treatment without differences between them, so the cited papers are consistent with ours.

Additionally, our study revealed strong positive correlations between MMP-8 and IL-8 concentrations in the experimental group, and moderate correlations in the control group. A significant reduction in IL-8 concentration was observed in both groups, indicating effective inflammation suppression and demonstrating the effectiveness of non-surgical periodontal treatment. However, the concentration of MMP-8 was only effectively reduced in the study group, indicating an additional positive effect of the gel used. A decrease in MMP-8 concentration could have long-term benefits as it could reduce the risk of periodontal tissue loss.

Our study is an in-depth clinical and molecular evaluation of the NaOCl/AA gel, but it has some limitations. The NaOCl/AA formulation was applied once; we cannot tell how multiple applications would work in the deep pockets. For molecular evaluation, we chose MMP-8 and IL-8 as representatives; howeverthe molecular panel could be expanded. Additionally, the only criterion for selecting pockets for GCF collection was their initial depth; we did not consider the type of tooth or its location. This may pose a risk of bias. Front teeth are easier to clean than multi-rooted teeth, which may indirectly affect the sample content.

## 5. Conclusions

Considering the above, a single application of the NaOCl/AA gel in deep periodontal pockets does not affect clinical results and IL-8 levels. However, it may have an effect on the amount of MMP-8, which (due to baseline differences) requires further verification.

## Figures and Tables

**Table 1:** Comparison of FMPS and FMBOP values before and after therapy.

	Baseline	3 Months	6 Months	*p*
FMPS (%)				
Test group (NaOCl/AA+ SRP)	28.56 ± 13.78	21.82 ± 10.73	19.73 ± 11.06	0.17
Control group (SRP)	26.9 ± 12.51	21.85 ± 10.53	24.40 ± 9.17	0.26
*p* *	0.61	0.99	0.07	
FMBOP (%)				
Test group (NaOCl/AA+ SRP)	28.53 ± 14.99	16.93 ± 7.32	14.32 ± 7.25	<0.001
Control group (SRP)	18.57 ± 8.24	14.44 ± 7.21	14.35 ± 6.75	0.02
*p* *	0.06	0.26	0.83	

FMPS—full mouth plaque score, FMBOP—full mouth bleeding on probing, *p*—Anova Friedman’s test, *p* *—U Mann–Whitney test.

**Table 2:** Mean concentrations of MMP-8 in ng/mL, IL-8 in pg/mL, and volume of fluid (SFFR) in relative Periotron units (PU) collected for examination from the chosen pocket (PD ≥ 5 mm) in the study and control group.

	Test Group NaOCl/AA + SRP	Control Group SRP	*p* *
MMP-8 (ng/mL)			
Baseline	8.32 ± 4.42	4.14 ± 3.85	<0.01
1 week	4.74 ± 5.26	6.50 ± 5.05	0.31
2 weeks	4.40 ± 4.40	6.86 ± 5.36	0.20
3 months	5.14 ± 5.26	4.81 ± 4.87	0.92
*p*	0.01	0.35	
IL-8 (pg/mL)			
Baseline	498.8 ± 126.4	444.1 ± 96	0.16
1 week	456.3 ± 112.3	465.3 ± 157.7	0.6
2 weeks	359.8 ± 89.7	371.5 ±193	0.44
3 months	325.9 ±101.4	352.7 ± 168.4	0.89
*p*	<0.0001	=0.0008	
SFFR (PU)			
Baseline	109.25 ± 37.06	112.70 ± 47.53	0.7
1 week	95.60 ± 43.47	95.30 ± 49.57	0.96
2 weeks	105.30 ± 49.49	114.05 ± 55.40	0.57
3 months	106.45 ± 51.11	87.70 ± 37.90	0.21
*p*	0.88	0.12	

MMP-8—metalloproteinase 8, IL-8—interleukin 8, SFFR—sulcus fluid flow rate, *p*—Anova Friedman’s test, *p* *—U Mann–Whitney’s test.

**Table 3:** Spearman’s rank order correlations in the three-month observation.

		Study Group *n* = 20		Control Group *n* = 20	
	Pair of Variables	R	*p*	R	*p*
Baseline	MMP-8 and SFFR	0.49	*p*= 0.03	ns	ns
	MMP-8 and IL-8	0.69	*p* = 0.0007	0.7	*p* = 0.0006
	IL-8 and SFFR	ns	ns	ns	ns
	*n* PD ≥ 5 mm and FMPS	ns	ns	ns	ns
	*n* PD ≥ 5 mm and FMBOP	ns	ns	0.62	*p* = 0.003
1 week	MMP-8 and SFFR	0.48	*p* = 0.03	ns	ns
	MMP-8 and IL-8	0.72	*p* = 0.0004	ns	ns
	IL-8 and SFFR	0.48	*p* = 0.03	ns	ns
2 weeks	MMP-8 and SFFR	ns	ns	ns	ns
	MMP-8 and IL-8	ns	ns	0.59	*p* = 0.005
	IL-8 and SFFR	ns	ns	ns	ns
3 months	MMP-8 and SFFR	ns	ns	0.46	*p* = 0.04
	MMP-8 and IL-8	0.82	*p* = 0.000007	0.57	*p* = 0.009
	IL-8 and SFFR	ns	ns	ns	ns
	*n* PD ≥ 5 mm and FMPS	ns	ns	ns	ns
	*n* PD ≥ 5 mm and FMBOP	ns	ns	0.51	p = 0.02

ns—non significant, R-Spearman’s correlation coefficient, *n* PD ≥ 5 mm—number of pockets PD ≥ 5 mm.

## Data Availability

The original contributions presented in the study are included in the article, further inquiries can be directed to the corresponding author.

## Abbreviations

The following abbreviations are used in this manuscript:

NaOCl/AA	Sodium hypochlorite amino acid gel
MMP-8	Metalloproteinase 8
IL-8	Interleukin 8
GCF	Gingival Crevicular Fluid
PD	Pocket depth
CAL	Clinical attachment level
GR	Gingival recession

## Appendix A

**Table A1:** Comparison of average periodontal parameters such as probing depth (PD), gingival recession (GR), and clinical attachment level (CAL) for all examined teeth in patients before and after treatment in both groups.

	Baseline	3 Months	6 Months	*p*	Diff (0–6 m)
PD mean (mm)					
Test group	3.51 ± 0.76	3.14 ± 0.71	2.85 ± 0.49	<0.001	0.67 ± 0.38
Control group	3.20 ± 0.44	2.81 ± 0.43	2.63 ± 0.35	<0.001	0.57 ± 0.33
*p* *	0.39	0.22	0.22		
GR mean (mm)					
Test group	0.58 ± 0.54	0.58 ± 0.52	0.63 ± 0.52	0.30	−0.05 ± 0.17
Control group	0.78 ± 0.98	0.61 ± 0.71	0.75 ± 0.82	0.41	0.03 ± 0.23
*p* *	0.84	0.75	0.95		
CAL mean (mm)					
Test group	4.10 ± 1.05	3.76 ± 1.04	3.50 ± 0.78	<0.001	0.6 ± 0.47
Control group	3.71 ± 1.15	3.35 ± 0.76	3.55 ± 1.51	<0.001	0.15 ± 2.14
*p* *	0.66	0.24	0.34		

*p*—Anova Friedman test, *p* *—U Manna-Whitney test.

**Table A2:** Comparison of changes in mean PD, GR, and CAL values for pockets with an initial PD value ≥ 5 mm before and after periodontal therapy.

	Baseline	3 Months	6 Months	*p*	Diff. 0–6 m
PD mean(mm)					
Test group	6.02 ± 0.66	4.85 ± 0.81	4.23 ± 0.67	<0.001	1.80 ± 0.49
Control group	5.75 ± 0.52	4.60 ±0.74	4.18 ± 0.68	<0.001	1.57 ± 0.64
*p* *	0.26	0.48	0.97		
GR mean (mm)					
Test group	0.74 ± 0.78	0.75 ± 0.72	0.83 ± 0.78	0.07	−0.1 ± 0.67
Conntrol group	0.51 ± 0.73	0.54 ± 0.70	0.65 ± 0.79	<0.01	−0.14 ± 0.2
*p* *	0.09	0.12	0.23		
CAL mean (mm)					
Test group	6.76 ± 1.13	5.60 ± 1.28	5.06 ± 1.16	<0.001	1.7 ± 0.59
Control group	6.27 ± 0.98	5.14 ± 1.04	4.83 ± 1.12	<0.001	1.43 ± 0.72
*p* *	0.20	0.40	0.99		

PD—probing depth, GR—gingival recession, CAL—clinical attachment level, *p*—Anova Friedman test, *p* *—U Manna Whitney test.

**Table A3:** Changes in periodontal parameters (PD, GR, CAL) of pocket with PD ≥ 5 mm selected for laboratory testing during the first visit.

	Baseline	3 Months	6 Months	*p*
PD mean (mm)				
Test group	6.95 ± 1.88	5.20 ± 2.21	4.45 ± 1.73	<0.001
Control group	6.20 ± 0.95	5.30 ± 1.49	4.80 ± 1.47	<0.001
*p* *	0.30	0.36	0.16	
GR mean (mm)				
Test group	1.65 ± 1.81	1.60 ± 1.67	1.65 ±1.42	0.95
Control group	0.45 ± 0.94	0.35 ± 0.75	0.45 ± 0.89	0.58
*p* *	0.02	<0.01	<0.01	
CAL mean (mm)				
Test group	8.60 ± 3.14	6.80 ± 3.22	6.10 ± 2.63	<0.001
Control group	6.65 ± 1.22	5.70 ± 1.81	5.25 ± 1.86	<0.001
*p* *	0.047	0.38	0.39	

PD—probing depth, GR—gingival recession, CAL—clinical attachment level, *p*—Anova Friedman test, *p* *—U Manna-Whitney test.
